# HeraNorm: an R shiny application for identifying optimal endogenous controls for miRNA and RNA assays in qPCR and ddPCR

**DOI:** 10.1093/bioadv/vbaf220

**Published:** 2025-09-17

**Authors:** Yao Hu, Xiaochun Xu, Yirong Shen, Liang You, Yanqin Yu, Libo Zhu, Farideh Bischoff, Xinmei Zhang, Wing Hing Wong

**Affiliations:** HerAnova Lifesciences, Hangzhou, 310018, China; HerAnova Lifesciences, Hangzhou, 310018, China; HerAnova Lifesciences, Hangzhou, 310018, China; HerAnova Lifesciences, Hangzhou, 310018, China; HerAnova Lifesciences, Hangzhou, 310018, China; Women’s Hospital, School of Medicine, Zhejiang University, Hangzhou, 310006, China; HerAnova Lifesciences, Burlington, MA 01803, United States; Women’s Hospital, School of Medicine, Zhejiang University, Hangzhou, 310006, China; HerAnova Lifesciences, Burlington, MA 01803, United States

## Abstract

**Motivation:**

Normalization is a critical step in quantitative PCR (qPCR) and droplet digital PCR (ddPCR) experiments to ensure accurate and reproducible gene expression analysis. However, commonly used endogenous controls such as miR-16 for miRNA assays and GAPDH for RNA assays have been shown to be unsuitable in certain disease conditions due to expression variability.

**Results:**

Here, we present a R Shiny application designed to identify the most stable endogenous controls specific to a given dataset or disease. Our interactive tool enables researchers to select optimal reference genes that can enhance the reliability of miRNA and RNA quantification in routine clinical diagnostic tools such as qPCR and ddPCR.

**Availability and implementation:**

HeraNorm is implemented in R and is available at https://github.com/Heranova-Lifesciences/HeraNorm.

## 1 Introduction

Accurate gene expression analysis is a cornerstone of modern clinical diagnostics and biomedical research, underpinning critical applications such as cancer subtyping, early detection of diseases, and monitoring treatment responses (Bustin 2024). While next-generation sequencing (NGS) has transformed biomarker discovery by enabling unbiased, genome-wide surveys of transcriptional and genomic alterations, its reliance on sophisticated bioinformatic infrastructure, lengthy data-processing pipelines, and high costs (often exceeding $1000 per sample for RNA-Seq depending on sequencing depth) limit its utility in routine clinical practice (Goodwin *et al.* 2016). In contrast, quantitative polymerase chain reaction (qPCR) and increasingly, droplet digital PCR (ddPCR) remain the standard practice in diagnostic settings due to their affordability ($2–$50 per reaction), rapid turnaround (<2 h), and adaptability to high-throughput workflows (Huggett *et al.* 2015, Taylor *et al.* 2017, Vandenberg *et al.* 2021). These methods have been indispensable for quantifying viral loads (e.g. SARS-CoV-2), and validating non-coding RNAs (such as microRNAs or miRNAs) as diagnostic as well as prognostic markers (Miotto *et al.* 2014, Bidarra *et al.* 2019, Vandenberg *et al.* 2021).

A critical barrier to translating NGS-derived biomarkers into clinically actionable PCR assays lies in the flawed selection of endogenous controls (ECs) for data normalization. Unlike NGS, which uses global normalization strategies (e.g. reads per kilobase million [RPKM], transcripts per million [TPM], or algorithmic approaches like DESeq2), qPCR and ddPCR rely on target-specific normalization using endogenous reference genes to account for technical variabilities (e.g. RNA input, reverse transcription efficiency) and biological heterogeneity (e.g. tumor cellularity, tissue type) (Chapman and Waldenstrom 2015, Taylor *et al.* 2019). However, many studies directly default to “universal” ECs (e.g. GAPDH, TBP, RNU48, or miR-16) without validating their stability in the specific disease context or assay platform. This oversight introduces systematic bias, rendering biomarker quantification unreliable and irreproducible in clinical settings (Chapman and Waldenstrom 2015, Taylor *et al.* 2019). This concern is particular critical when dealing with circulating small RNAs, such as miRNAs, which are gaining prominence as promising biomarkers in clinical diagnostics across a wide range of diseases, including cancers (Metcalf 2024), infectious diseases (Grehl *et al.* 2021), and neurodegenerative disorders (Sheinerman *et al.* 2017). In this context, the inappropriate selection or use of endogenous controls for normalization can introduce significant bias, leading to erroneous results, compromised data interpretation and poor reproducibility across studies (Hirschberger *et al.* 2019, Madadi *et al.* 2019). For example, commonly used reference genes such as RNU44 and RNU48 in miRNA studies yielded erroneous results in breast cancer and head and neck squamous cell carcinoma due to the reference genes correlating with tumor pathology and patient diagnosis (Gee *et al.* 2011). Similarly, miR-16, frequently used as a reference in miRNA-based studies, has been shown to correlate with technical variables rather than biological signals, and may not be suitable as a reference marker in serum samples due to potential hemolysis (Pritchard *et al.* 2012). Moreover, miR-16 has been found to correlate with disease progression in various diseases including melanoma and cardiovascular disease, potentially leading to inaccurate results if used as a reference (Friedman *et al.* 2012, Toro *et al.* 2022).

The repercussions of improper normalization extend beyond scientific inaccuracy, contributing to costly delays in diagnostic development and eroded confidence in precision medicine. To address this, recent guidelines, such as the MIQE (Minimum Information for Publication of Quantitative Real-Time PCR Experiments), recommend the empirical validation of reference genes using stability algorithms like geNorm (Vandesompele *et al.* 2002) or NormFinder (Andersen *et al.* 2004), which were originally developed for qPCR-based and microarray studies in the early 2000s. These tools evaluate candidate reference genes by analyzing expression stability across sample cohorts, with geNorm ranking genes based on pairwise variation and NormFinder modeling intra- and inter-group variability. While transformative for qPCR workflows in the early 2000s, their design inherently limits their applicability in modern biomarker discovery pipelines, where new biomarkers and ECs can now be identified from large NGS datasets to guide downstream qPCR or ddPCR assays. Both geNorm and NormFinder are tailored for smaller-scale studies, typically involving approximately 10 candidates in a qPCR-based or microarray arrays. The original implementation was in Excel using macros, with more recent versions in R or Python. This framework is typically not easily amendable to NGS discovery datasets, which profile thousands of genes or miRNAs across hundreds of samples (Grabia *et al.* 2020). These older tools also lack visualization capabilities and *in silico* testings for normalization using data afforded by NGS-based pipeline. For researchers aiming to transition from NGS biomarker discovery to PCR-based validation, these limitations create a disjointed workflow, hindering the identification of robust ECs validated across platforms.

To bridge the technical gap between NGS-based biomarker discovery and clinical diagnostic implementation, we developed an R Shiny app that facilitates the systematic identification and validation of endogenous controls (ECs) from NGS datasets, ensuring reliable translation to qPCR/ddPCR assays. The app uses a wrapper around DESeq2 (Love *et al.* 2014), integrating median-of-ratios normalization and negative binomial modeling to resolve overdispersion and compositional biases in NGS-based RNA-seq and miRNA-seq data, overcoming limitations of legacy tools like geNorm and NormFinder. Users can directly upload raw count matrices—such as outputs from RSEM or HTseq for RNA-seq (Li and Dewey 2011, Anders *et al.* 2015), or miRge3 for miRNA-seq (Patil and Halushka 2021). The app includes modules that enable visualization of EC candidates and also differentially expressed biomarkers via heatmaps and boxplots, and *in silico* simulation of qPCR outcomes by normalizing user-selected biomarkers against app-identified ECs. The app outputs stability rankings, directly linking NGS discovery to clinical assay design.

## 2 Implementation

### 2.1 Data input and processing

The application requires two input files: (i) a count matrix (genes/miRNAs as rows, samples as columns) and (ii) a metadata table (samples as rows, experimental variables as columns). The count matrix can be generated from standard RNA-Seq or miRNA-Seq pipelines, such as RSEM or HTSeq for RNA-Seq, or miRge3 and sRNAtoolbox for miRNA-Seq. Example templates for both files are provided on the GitHub repository, ensuring proper formatting (e.g. gene identifiers, sample names).

**Figure 1.**
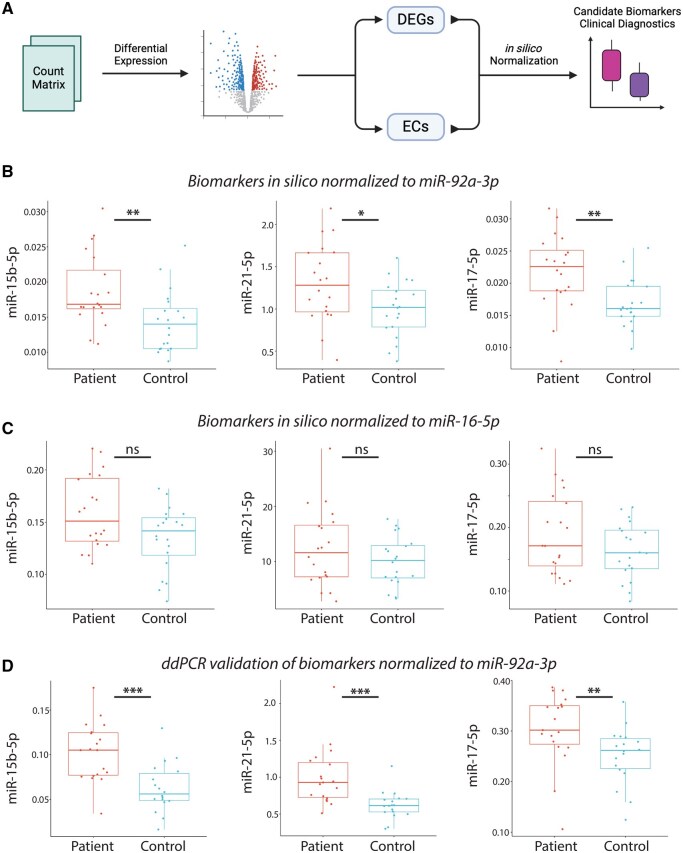
HeraNorm for identifying candidate endogenous controls in clinical diagnostics. (A) The HeraNorm pipeline processes a count matrix to perform differential expression analysis, identifying both differentially expressed genes (DEGs) and potential endogenous controls. The app also enables *in silico* normalization of DEGs using the identified endogenous controls. This panel was constructed in Biorender.com (B) Normalizing DEGs to the app-identified endogenous control, miR-92a-3p, reveals a significant difference between patient and control groups in the NGS discovery dataset. (C) In contrast, normalizing DEGs to the commonly used universal control, miR-16-5p, fails to show a significant difference between patient and control groups in the NGS discovery dataset. (D) Experimental validation using ddPCR, with miR-92a-3p as the endogenous control, replicates the findings observed in the NGS discovery cohort. The *y*-axis represents the ratio of DEG counts to endogenous control counts. * denotes *P*-value ≤ .05; ** denotes *P*-value ≤ .01; *** denotes *P*-value ≤ .001. Pairwise comparisons were performed using Wilcoxon rank-sum test.

Through an intuitive graphical user interface (GUI), users upload these files and initiate analysis with default parameters or custom settings. The app will then pre-process the raw count matrix, implementing median-of-ratios normalization to correct for library size and compositional biases. Differential expression analysis is performed via negative binomial regression model, incorporating user-defined sample groups (e.g. disease versus control) from the metadata. This step estimates log2 fold changes (log2FC), *P*-values and false discovery rates (FDR) for all genes or miRNAs (Fig. 1A).

### 2.2 Identification of context-specific endogenous controls (ECs) and biomarkers

Differentially Expressed Biomarker Selection: Differentially expressed genes (DEGs) or miRNAs are ranked by statistical significance (*P*-value ≤ .05 by default) and log2FC magnitude. Users can adjust thresholds (e.g. *P*-value ≤ .01, |log2FC| > 1) to prioritize high-impact candidates. A curated list of biomarkers is generated, highlighting transcripts with robust differential expression.

EC Identification: To select ECs, the app evaluates expression stability using dispersion estimates and log2FC constraints (default: |log2FC| < 0.02 between groups). Candidates with minimal intra- and inter-group variability are retained based on *P*-values ≥ .8 (users can adjust the threshold according to their respective study design; recommended values are at least ≥ 0.6), ensuring they are minimally affected by experimental conditions. Users can modify these thresholds as needed. Final biomarker and EC lists are exportable as CSV files for downstream assessments.

### 2.3 Visualization of *in silico* normalization for qPCR and ddPCR

The app generates visualizations to assess biomarkers and ECs performance. Heatmaps and volcano plots display expression patterns of top DEGs across sample groups, while boxplots illustrate their distributional differences. Statistical validation is further reinforced through Wilcoxon rank-sum tests, which evaluate group differences independent of the negative-binomial testing.

To enhance translational relevance, the app models qPCR/ddPCR workflows by normalizing differentially expressed biomarkers to user-selected endogenous controls (ECs). This *in silico* normalization calculates the ratio of biomarker expression to EC expression, which can be directly compared to ratio-based ddPCR calculations. A higher ratio indicates greater biomarker expression relative to the EC expression. Although qPCR delta CT calculations differ—where a lower delta CT indicates higher expression—users can easily adjust the observations between groups by flipping the effect direction, as visualized in the generated boxplots.

### 2.4 Benchmarking assessments

To validate the app’s utility, we first analyzed a miRNA-Seq dataset generated in-house comprising serum samples from 20 endometriosis patients and 20 non-disease controls (Yu *et al.* 2025). Differential expression analysis identified 85 differentially expressed miRNAs, including miR-21-5p (upregulated) and miR-17-5p (upregulated). The app nominated several EC candidates such as miR-92a-3p and miR-421 (*P*-value ≥ .8, |log2FC| ≤ 0.02); while excluding the commonly (mis)-used miR-16-5p which did not meet the criteria of a stable EC due to having larger |log2FC| and lower *P*-value than the thresholds (Fig. 1B and C). This underscores the risks of “universal” references like miR-16-5p and the app’s ability to identify context-stable ECs. We experimentally validated these findings: normalization of miR-21-5p to miR-92a-3p yielded significant differences between the two patient cohorts, aligning with NGS results (Fig. 1D).

To further demonstrate HeraNorm’s utility, we analyzed two additional datasets: an in-house RNAseq dataset comparing endometrial tissue from patients with and without endometriosis (patients = 18, controls = 18; available on the app GitHub page, and on Genome Sequence Archive with accession number PRJCA043935), and a miRNAseq dataset from a published study for non-invasive Creutzfeldt-Jakob disease biomarker discovery (patients = 57, controls = 48) (Norsworthy *et al.* 2020). In the endometrial RNAseq dataset, 654 genes were differentially expressed (*P*-value ≤ .05; average expression ≥ 100 normalized counts). RPS11 emerged as a stable endogenous control in this context (*P*-value > .8, log2FC = 0.012), consistent with prior reports of its stability across disease groups (Zhan *et al.* 2014, Sarcinelli *et al.* 2020). In contrast, canonical ECs like TBP (*P*-value < .1; log2FC = −0.11) and GAPDH (*P*-value < .7; log2FC = 0.036) showed higher variability, potentially confounding results. Among the top 10 differentially expressed genes, *in silico* normalization using RPS11 recapitulated 9/10 patterns observed in the NGS data, compared to only 4/10 and 5/10 for TBP and GAPDH respectively, highlighting the risk of errors when using default, universal ECs (Fig. 1, available as supplementary data at *Bioinformatics Advances* online).

In the Creutzfeldt-Jacob miRNAseq study (Norsworthy *et al.* 2020), previously reported differentially expressed miRNAs, namely miR-93-5p, miR-106b-3p and let-7i-5p were also identified in our independent re-analysis (Fig. 2, available as supplementary data at *Bioinformatics Advances* online). Notably, the original authors found that miR-16-5p, a commonly used endogenous control, was itself differentially expressed in this disease context. Using HeraNorm, we identified miR-186-5p (*P*-value = .76; log2FC = −0.06; average read count = 465) was a more stable EC (Belmonte *et al.* 2024). *In silico* normalization with miR-186-5p preserved the original findings by NGS, whereas normalization with miR-16-5p obscured them (Fig. 2, available as supplementary data at *Bioinformatics Advances* online). This underscores the risk of relying on canonical ECs without validation and highlights the importance of context-specific EC selection.

## 3 Discussion

The choice of endogenous controls (ECs) that maintain stability across varied biological and technical conditions continues to be a major challenge in gene expression analysis, significantly impacting diagnostic accuracy and research reproducibility. Conventional reference genes such as GAPDH, TBP, or miR-16-5p, frequently chosen based on historical usage rather than empirical evidence, show considerable variability in contexts like cancer, metabolic disorders, and biofluids prone to hemolysis. Our R Shiny application tackles this limitation by replacing heuristic approaches with a data-driven framework built on robust statistical models and workflows optimized for NGS. Using *in silico* normalization simulations, we identified a set of biomarker candidates and potential endogenous controls, which were experimentally validated. These findings were replicated in both qPCR and ddPCR assays, underscoring the tool’s effectiveness in bridging NGS-based discovery data with clinical assay development.

Future iterations of the software will integrate machine learning models to predict ECs stability across untested conditions, leveraging large-scale transcriptomic databases (e.g. TCGA, GTEx) to identify robust EC candidates with superior stability in specific disease context.

## 4 Conclusion

Our R Shiny application provides a solution to the persistent challenge of endogenous control selection in gene expression studies. By coupling a rigorous normalization framework with interactive, user-friendly visualization, the tool bridges the gap between NGS-driven biomarker discovery and clinical assay development. It eliminates reliance on unstable “universal” references, instead identifying context-stable ECs through statistical rigor—demonstrated in our endometriosis benchmarking, where app-selected ECs restored diagnostic significance lost when using miR-16. The software’s open-source architecture and compatibility with standard PCR workflows empower researchers to enhance data robustness across diverse applications, from cancer subtyping to infectious disease monitoring.

## Supplementary Material

vbaf220_Supplementary_Data

## Data Availability

The application is implemented in R Shiny and is freely available for download in the GitHub repository (https://github.com/Heranova-Lifesciences/HeraNorm). Source code and documentation are provided in the Github to facilitate customization and integration into existing pipelines.
