# Supplementary material for: HeraNorm: an R shiny application for identifying optimal endogenous controls for miRNA and RNA assays in qPCR and ddPCR
Source: Bioinform Adv. 2025 Sep 17;5(1):vbaf220. doi: 10.1093/bioadv/vbaf220 (PMC12596163; doi:10.1093/bioadv/vbaf220)
Supplement: vbaf220_Supplementary_Data [file vbaf220_supplementary_data.pdf]

Supplementary Materials

HeraNorm: a R Shiny application for identifying optimal endogenous controls for miRNA and RNA assays in qPCR and ddPCR.

Yao Hu[1], Xiaochun Xu[1], Yirong Shen[1], Liang You[1], Yanqin Yu[1], Libo Zhu[2], Farideh Bischoff[3], Xinmei Zhang[2,*], Wing Hing Wong[3,*]

[1]Heranova Lifesciences, Hangzhou, China

[2]Women's Hospital, School of Medicine, Zhejiang University, Hangzhou, China

[3]Heranova Lifesciences, Burlington, MA 01803, USA

*Corresponding authors:

Xinmei Zhang (zhangxinm@zju.edu.cn)

Wing Hing Wong (wing.h.wong@heranova.com)

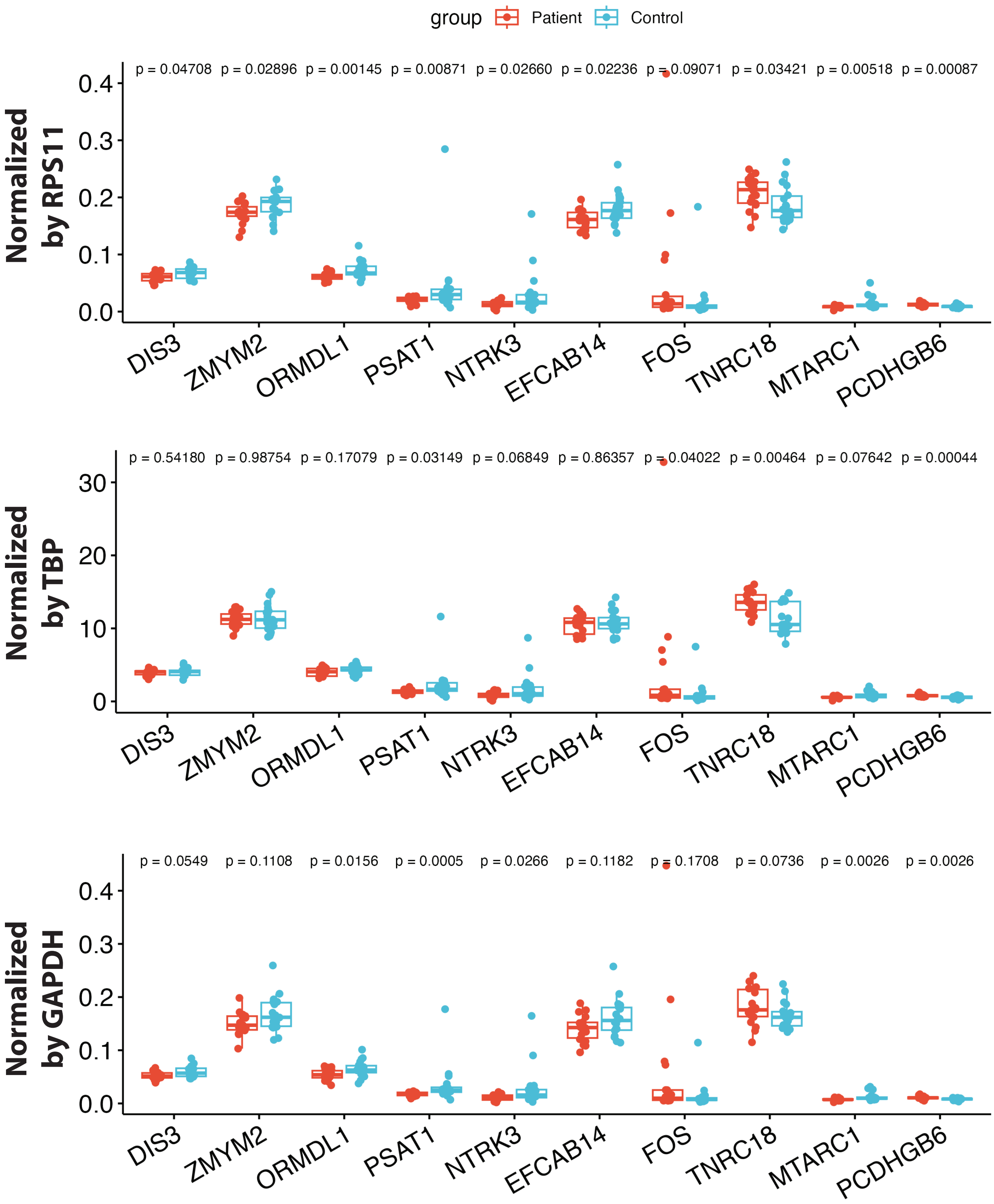

Supplementary Figure S1: In-house RNAseq data from endometrial tissues of endometriosis patients and controls were nomalized in silico using different endogenous controls (EC). HeraNorm identified RPS11 as a suitable control, with normalization closely matching NGS results for the top 10 DEGs (top panel). In contrast, normalization using canonical ECs (TBP and GAPDH) failed to replicate NGS findings for ≥50% of these top 10 DEGs. Pairwise comparisons were performed using Wilcoxon rank-sum test.

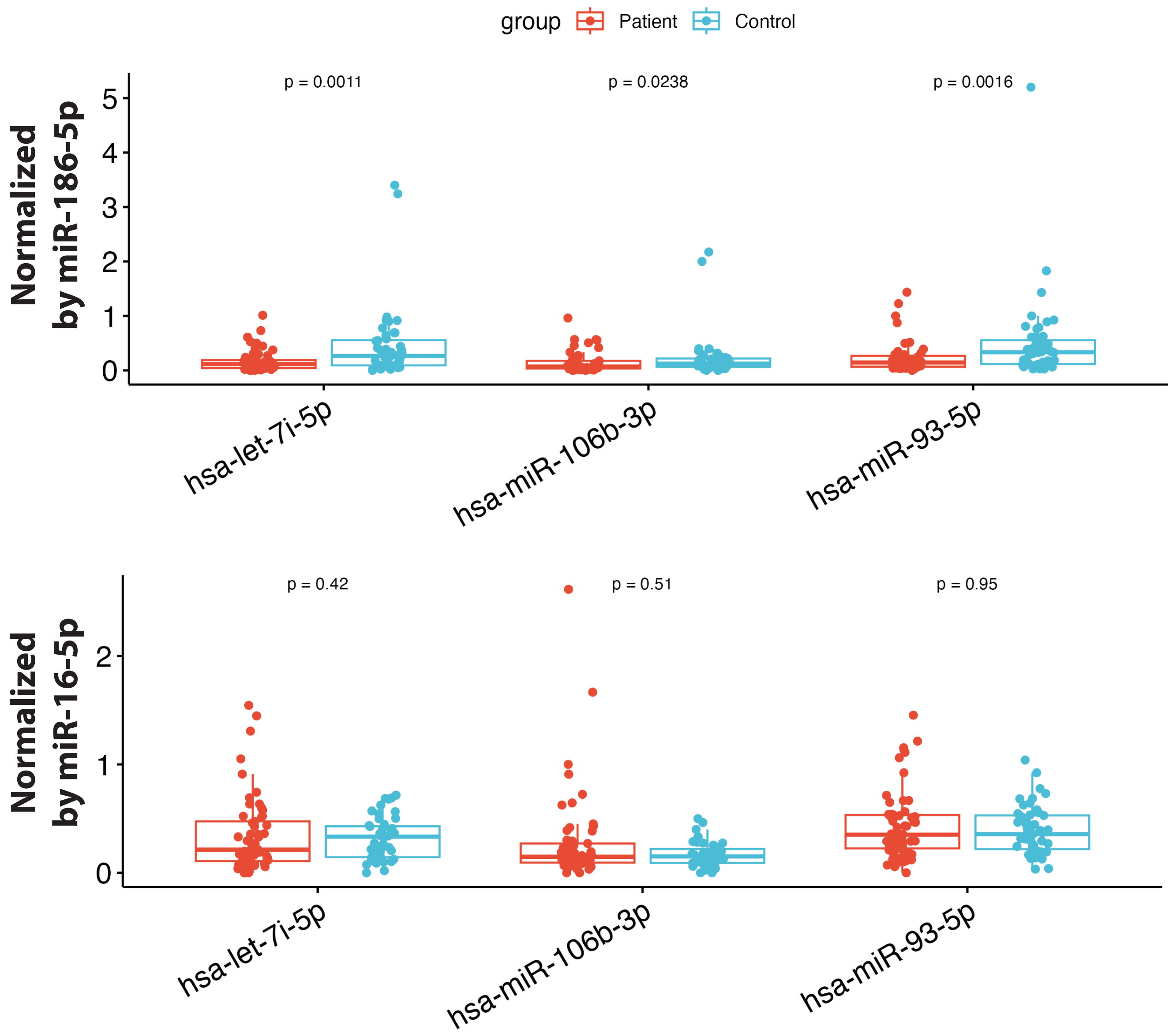

Supplementary Figure S2: Re-analysis of a published miRNAseq dataset on Creutzfeldt-Jakob disease biomarker discovery (Reference #27 in manuscript). The original study reported several differentially expressed miRNAs, including let-7i-5p, miR-106-3p and miR-93-5p, all of which were confirmed in our re-analysis. Notably, miR-16-5p, a commonly used endogenous control, was also reported as differentially expressed by both the original authors and our independent analysis. HeraNorm instead identified miR-186-5p as a stable control in this disease context, and in silico normalization using miR-186-5p successfully reproduced the NGS findings (top panel). In contrast, normalization using miR-16-5p failed to do so.
